# Validation of full-field optical coherence tomography in distinguishing malignant and benign tissue in resected pancreatic cancer specimens

**DOI:** 10.1371/journal.pone.0175862

**Published:** 2017-04-17

**Authors:** Labrinus van Manen, Paulien L. Stegehuis, Arantza Fariña-Sarasqueta, Lorraine M. de Haan, Jeroen Eggermont, Bert A. Bonsing, Hans Morreau, Boudewijn P. F. Lelieveldt, Cornelis J. H. van de Velde, Alexander L. Vahrmeijer, Jouke Dijkstra, J. Sven D. Mieog

**Affiliations:** 1 Department of Surgery, Leiden University Medical Center, Leiden, The Netherlands; 2 Division of Image Processing, Department of Radiology, Leiden University Medical Center, Leiden, The Netherlands; 3 Department of Pathology, Leiden University Medical Center, Leiden, The Netherlands; Tufts University, UNITED STATES

## Abstract

**Background:**

Pancreatic cancer is the fourth leading cause of cancer-related mortality in the United States. The minority of patients can undergo curative-intended surgical therapy due to progressive disease stage at time of diagnosis. Nonetheless, tumor involvement of surgical margins is seen in up to 70% of resections, being a strong negative prognostic factor. Real-time intraoperative imaging modalities may aid surgeons to obtain tumor-free resection margins. Full-field optical coherence tomography (FF-OCT) is a promising diagnostic tool using high-resolution white-light interference microscopy without tissue processing. Therefore, we composed an atlas of FF-OCT images of malignant and benign pancreatic tissue, and investigated the accuracy with which the pathologists could distinguish these.

**Materials and methods:**

One hundred FF-OCT images were collected from specimens of 29 patients who underwent pancreatic resection for various indications between 2014 and 2016. One experienced gastrointestinal pathologist and one pathologist in training scored independently the FF-OCT images as malignant or benign blinded to the final pathology conclusion. Results were compared to those obtained with standard hematoxylin and eosin (H&E) slides.

**Results:**

Overall, combined test characteristics of both pathologists showed a sensitivity of 72%, specificity of 74%, positive predictive value of 69%, negative predictive value of 79% and an overall accuracy of 73%. In the subset of pancreatic ductal adenocarcinoma patients, 97% of the FF-OCT images (n = 35) were interpreted as tumor by at least one pathologist. Moreover, normal pancreatic tissue was recognised in all cases by at least one pathologist. However, atrophy and fibrosis, serous cystadenoma and neuroendocrine tumors were more often wrongly scored, in 63%, 100% and 25% respectively.

**Conclusion:**

FF-OCT could distinguish normal pancreatic tissue from pathologic pancreatic tissue in both processed as non-processed specimens using architectural features. The accuracy in pancreatic ductal adenocarcinoma is promising and warrants further evaluation using improved assessment criteria.

## Introduction

Pancreatic cancer is the fourth leading cause of cancer related deaths with a 5-year survival rate of 8% in the United States [1]. Many patients present with locally advanced or metastasized disease and are beyond cure. Patients diagnosed with localized disease can undergo a potentially curable treatment and have a 5-year survival rate around 20% [2–4]. Treatment consists of high-risk surgery (morbidity of 40–50% and mortality around 5% [5, 6]) usually followed or preceded by chemo(radio)therapy. During surgery, it is important to achieve a complete (R0) resection, as a distance of the tumor from the resection margin of ≤1 mm (R1 resection) is one of the most important prognosticators for poor survival in Europe [2, 7–9]. However, adequate intraoperative judgment is challenging, because the surgeon has to rely on visual inspection and palpation only. The presence of peritumoral inflammation in pancreatic cancer makes the distinction with normal tissue even more difficult. The incidence of R1 resections is up to 60–80% in published series of standardized pathological assessment [8–13], underlining the importance of adequate intraoperative resection margin assessment.

Although various imaging modalities (such as CT, MRI, EUS and PET) are used to diagnose pancreatic cancer and assess its resectability prior to surgery, only few techniques are suitable during surgery [14]. Frozen section analysis is currently the most used intraoperative modality, especially to assess extra-pancreatic lesions [15]. However, it has low sensitivity (38%) evaluating resection margins, and is therefore only used in selected cases at our institution [15]. Intraoperative ultrasound can be used to detect metastases or assess resectability, but its value in reducing positive resection margins is unknown, [14] and is the study object of an ongoing clinical trial at our institution. An emerging technique is near-infrared fluorescence imaging, which is based on a fluorescent tracer and dedicated cameras. Pre-clinical results demarcating pancreatic tumor are promising, but use tracers that are currently not yet FDA approved [16]. Thus, the current state-of-the art surgical approach encompasses high-risk intervention with limited success and little to no intraoperative visualisation of the malignant process. Novel intraoperative imaging tools are needed to improve the assessment of resectability and to guide subsequent resection.

Optical coherence tomography (OCT) was first described in 1991 and uses low-coherence interferometry to produce 2-dimensional cross-sectional images [17]. It is already standard-of-care in ophthalmology and cardiology, and with the development of new OCT modalities, other fields of interest—like oncology—are being discovered [18]. Full-field OCT (FF-OCT) is such a newly developed modality, based on the principles of white light interference microscopy, and acquires *en face* images by illuminating the whole field of view without scanning [19]. It enables non-invasive high-resolution imaging up to several millimetres of tissue without the need for tissue processing, by measuring the backscattered light of tissue structures with different refractive indices. Several studies report the use of FF-OCT in the field of oncology, i.e. on ovarian [20], skin [21] and brain tissue [22]. All showed encouraging results; architectural changes could be identified, and in a quick fashion a large surface could be scanned. Only one study has been conducted in pancreatic cancer, but this was to evaluate fine needle aspirates [23]. FF-OCT has not yet been tested in pancreas resection specimens to discriminate malignant and benign tissues.

In this feasibility study, we investigated whether pathologists were able to distinguish malignant from normal and benign pancreatic tissue based on FF-OCT images, obtained from surgical specimens.

## Materials and methods

### Patient and sample selection

Twenty-nine patients who underwent surgery for suspect pancreatic cancer at the Leiden University Medical Center (LUMC) were included in this study. Fresh tissue samples were collected prospectively (October 2015 until January 2016) from surgical specimens from 17 patients with (pre)malignant pancreatic lesions. Formalin-fixed paraffin embedded (FFPE) samples from 12 patients were collected retrospectively (January 2014 until December 2014) from both benign and malignant pancreatic neoplasms to acquire FF-OCT images of different pancreatic neoplasms. No patients with a preoperative diagnosis of benign disease were included, for instance, patients with chronic or auto-immune pancreatitis were not included. From each of the 29 patients, if achievable, minimal one tissue section with presence of tumor and one tissue section without tumor were obtained. For the fresh samples, the selection was based on the macroscopic assessment by the pathologist; for FFPE samples, the selection was based on microscopic assessment. This resulted in 50 tissue samples (25 fresh samples and 25 FFPE samples). Per tissue sample, two regions of interest were selected by the study coordinator, LM, based on the corresponding H&E slides. These two regions of interest from a single tissue sections could be either both benign, both malignant or one malignant and one benign.

FFPE tissue blocks were deparaffinised, using a standard protocol. The study protocol was approved by the local medical ethics committee of the LUMC. The prospective collection of the fresh tissue samples was performed within the framework of routine clinical care. Therefore patient consent was not obtained, as this study was not subject to the Dutch Medical Research Involving Human Subjects Act, according to our local medical ethics committee. One author (LM) had access to patient information during clinical data acquisition after which patient data were anonymized. All patient samples and clinical data were handled in accordance with the medical ethics guidelines described in the Code of Conduct for the Proper Secondary Use of Human Tissue of the Dutch Federation of Biomedical Scientific Societies [24].

### FF-OCT imaging and sample processing

Images were obtained using a high resolution FF-OCT system (Light-CT^™^ scanner, LLTech SAS, Paris, France). In short, the setup consists of an upright microscope with a 10x objective, a halogen light-source with wavelength of 700±125 nm, and a reference arm in Linnik interferometric configuration [25, 26]. It generates high resolution (1.5 μm isotropic), 0.8 by 0.8 mm *en face* images, but the field of view is increased using image mosaicking (with a maximum diameter of 25 mm), at an image rate of 35 Hz. Image depth is adjustable, with a maximum depth of several millimeters, depending on tissue properties [27].

Tissue samples were placed in a sample holder in 0.9% NaCl solution with the surface to be imaged facing upward. A glass slide was positioned above the tissue to which it was gently flattened, and a layer of silicone oil was applied between the optical window and the microscope objective. A macroscopic image was obtained using a wide-field camera, followed by FF-OCT images. To ensure good correspondence with the histology images, FF-OCT images were acquired at a depth of around 20 μm. Imaging time for one sample was on average 30 minutes. After FF-OCT imaging, tissue samples were formalin fixed and embedded in paraffin. H&E slides were obtained and digitalised with a digital pathology slide scanner (IntelliSite Ultra Fast Scanner, Philips, Eindhoven, the Netherlands). FF-OCT images were viewed using in-house developed analysis software based on MeVisLab (MeVis Medical Solutions AG and Fraunhofer MEVIS, Germany).

### Study design

Two pathologists—one experienced and one resident—followed a brief training prior to assessment of the FF-OCT images. The training was divided in two parts: in the first part the technique and method of tissue imaging were explained, and in the second part FF-OCT images were shown with their corresponding H&E images. The images of this training atlas were magnified regions of interest showing details of the different pancreatic tissues (such as normal pancreas, pancreatitis and pancreatic ductal adenocarcinoma (PDAC)); the whole slide was not presented to the pathologists. Moreover, the regions of interest used in the training atlas were different in the test cohort.

**Fig 1:**
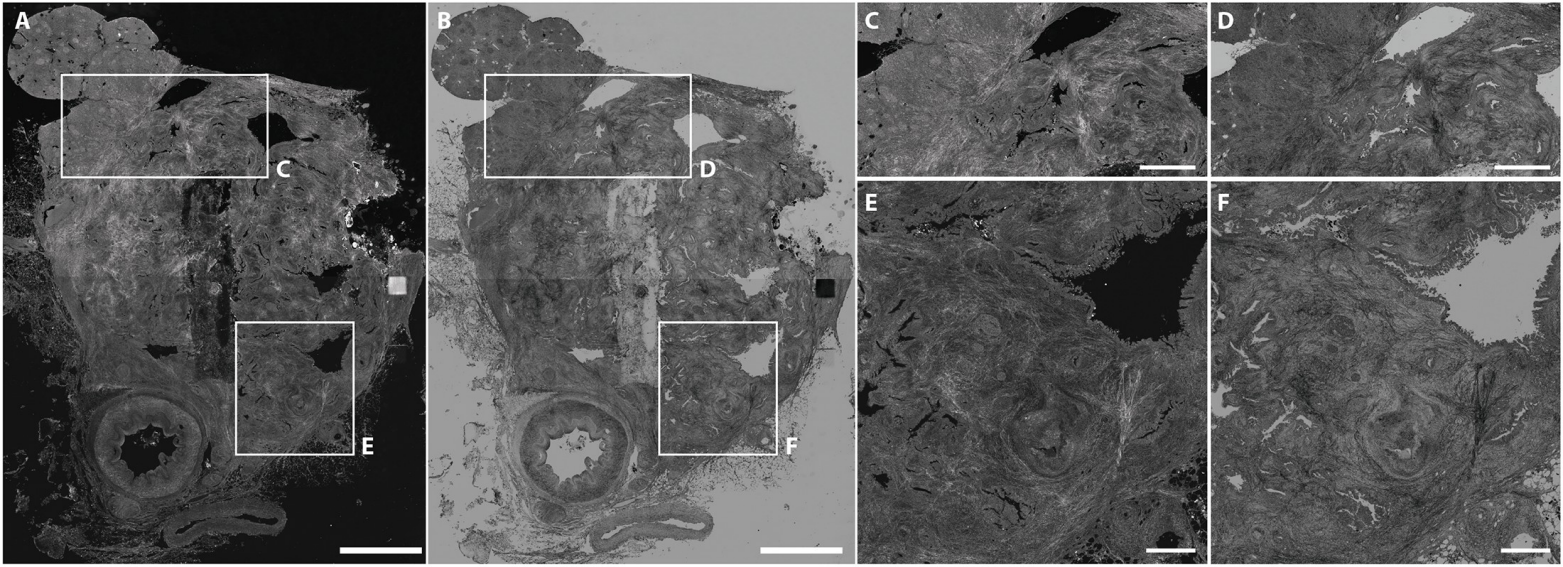
Example FF-OCT image of a well-differentiated pancreatic ductal adenocarcinoma as shown to the pathologist for assessment. The whole FF-OCT images were shown to the pathologists, but they were asked to only assess the selected regions of interest (two per FF-OCT image). Both the original (A,C,E) and the inverse (B,D,F) FF-OCT images are shown. Scale bars, 5 mm (A,B), 2 mm (C,D), and 1 mm (E,F).

The 100 regions of interest to be assessed by the pathologists were shown in the context of both the total FF-OCT image, and an enlarged (detail) image. They were offered to the pathologists in both the original and inverse setting, as shown in Fig 1. During assessment pathologists were able to digitally zoom the images. FF-OCT images were randomly presented in the same order to the pathologists who were blinded to the H&E slides, patient information and final pathological diagnosis of the specimen. The pathologists had to classify the marked region-of-interest as malignant or benign. If the pathologists found that the image quality was too poor to reach a decision, it was classified as not interpretable.

### Statistical analysis

The results of the individual region-of-interest scores were put in a 2x2 contingency table to calculate sensitivity, specificity, positive predictive value (PPV), negative predictive value (NPV) and accuracy for the complete cohort. In addition, diagnostic test characteristics and the interobserver variability were calculated after each cohort of 25 consecutive FF-OCT images. SPSS version 23 (IBM Corporation, Armonk, NY, USA) was used to calculate the interobserver variability and to compare the agreement in tumor identification by both pathologists, for which chi-squared tests were used. Kappa values were interpreted as described by Landis et Koch [28]. P<0.05 was considered statistical significant. Graphs were created using Graphpad version 7 (Graphpad Software, La Jolla, CA, USA).

## Results

**Fig 2:**
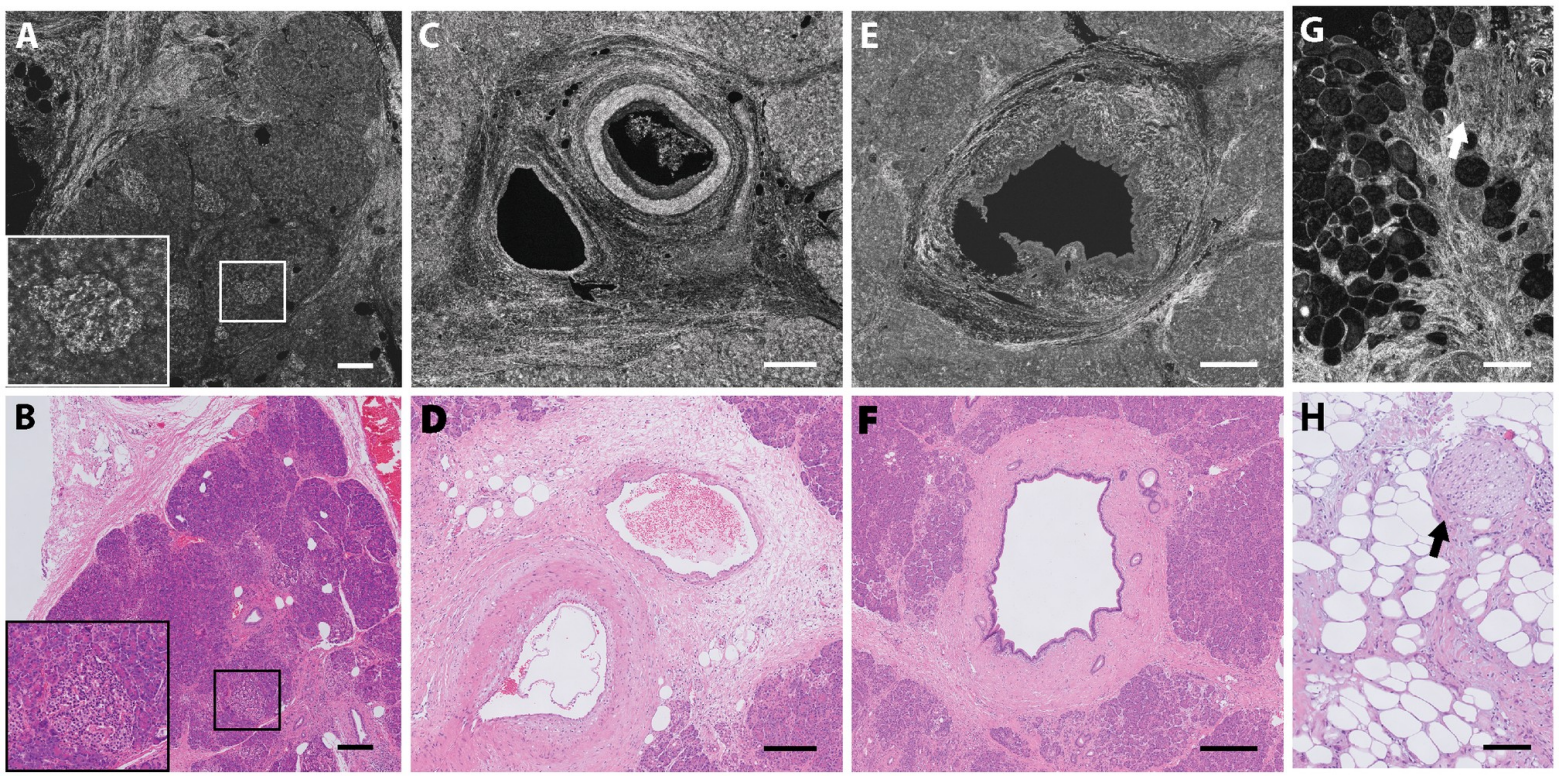
Examples of FF-OCT images of normal pancreatic tissue and corresponding histology. Structures that are easily identified on FF-OCT images include normal pancreatic parenchyma (A-B), vessels (C-D), and a large pancreatic duct (E-F). Harder to recognize are nerve bundles (G-H). Scale bars, 250 μm (A-B, G-H), and 150 μm (C-F). Inset shows an islet of Langerhans (A-B) at 2.5 times higher magnification.

In FF-OCT images of normal pancreatic tissue, the morphologic features of major components could be identified, like interlobular septae, acinar tissue, islet cells, pancreatic ducts and blood vessels (Fig 2). Recognition of nerve bundles in normal pancreatic tissue was difficult (Fig 2). Interlobular septae are visible as light grey strands which are located between the lobuli. Islet cells were recognised as highlighted groups of cells located in acinar tissue.

Histomorphology of pancreatic cancer in general and especially of PDAC is complex, being sometimes challenging to distinguish between malignant and reactive, benign glands in the context of pancreatitis even on H&E images [29]. On H&E images various criteria are used to distinguish between benign and malignant tissue. First of all, the pathologists look at low magnification level to detect the presence of normal pancreatic architecture. Desmoplastic stroma, irregular ducts and disorganised glands are characteristics that indicate potential malignancy. Atypical cell nuclei, intraluminal necrosis, perineural invasion and ingrowth into structures as lymph nodes, blood vessels and fat tissue are all characteristics that point toward malignancy. If the lobular structure is maintained even in the presence of irregular ducts with some cytonuclear atypia, pancreatitis should be considered. However, the difficulty resides in the fact that these essential features for the diagnostic on H&E slides are regularly not evaluable on the FF-OCT images. As shown in Table 1, only stroma and architectural distortion of pancreatic tissue and tumor glands are easily recognizable on FF-OCT images.

**Table 1:** Comparison of features of pancreatic ductal adenocarcinoma detectable on H&E and on FF-OCT images.

Features of malignancy	Detectable on H&E^a^	Detectable on FF-OCT
Disorganisation of lobuli and glands	+	+
Presence of atypical glands	+	±^b^
Atypical cell nuclei	+	-
Presence of tumor stroma	+	+
Ingrowth into structures (a.o. lymph nodes, blood vessels, fat)	+	±
Intraluminal necrosis	+	±
Perineural invasion	+	±

^**a**^ Adapted from Hruban et al, these diagnostic characteristics are used in assessment of H&E images.

^b^ Large atypical glands could be detected, but smaller glands are less visible and sometimes mistaken for blood vessels.

### Scoring FF-OCT images

Fifty samples from 29 patients have been included in this study. Patient and tumor characteristics are presented in Table 2. Of these tissue samples, 100 FF-OCT images were available for assessment: 57 benign and 43 malignant sections. The results of the FF-OCT assessment by the pathologists are detailed in Table 3 (and in S1 File). The more experienced gastrointestinal pathologist scored 6 images as not interpretable, the pathologist in training none. Of these 6 images– 3 fresh and 3 deparaffinised FFPE– 3 were benign and 3 were malignant. These images were scored by the pathologist in training; 3 images were scored correctly, and 3 images were scored incorrectly. Leaving these 6 images out of the analysis, the experienced pathologist achieved a higher accuracy than the less experienced pathologist: 80% versus 67%, respectively. The combined results showed the following test characteristics: sensitivity of 72%, specificity of 74%, PPV of 67%, NPV of 79% and an accuracy of 73%.

**Table 2:** Patient and tumor characteristics.

Characteristics	
**Age, *mean* (y)**	65.7
**Sex, n (%)**	
Male	13 (45)
Female	16 (55)
**ASA score, *n* (%)**	
1	5 (17)
2	19 (66)
≥3	5 (17)
**Tumor size (mm), *mean***	33.2
**Tumor location, n (%)**	
Pancreatic head	13 (45)
Pancreas body/tail	10 (35)
Distal CBD	1 (3)
Peri-ampullar	5 (17)
**Histological diagnosis, n (%)**	
PDAC	23 (79)
Well differentiated	3
Moderately differentiated	10
Poorly differentiated	7
Unknown^a^	3
IPMN	1 (3)
MCN	2 (7)
NET	2 (7)
Serous cystadenoma	1 (3)
**Surgical procedure, n (%)**	
PPPD	17 (59)
Whipple	1 (3)
Distal pancreatectomy	9 (31)
Central pancreatectomy	1 (3)
Total pancreatectomy	1 (3)

^a^ Differentiation grade could not be determined in 3 cases, because of pancreatic fibrosis after neoadjuvant (chemo)radiotherapy.

Abbreviations: ASA: American Society of Anesthesiologists; CBD: Common bile duct; PDAC: Pancreatic ductal adenocarcinoma; IPMN: Intraductal papillary mucinous neoplasm; MCN: Mucinous cystic neoplasm; NET: Neuroendocrine tumor; PPPD: Pylorus-preserving pancreaticoduodenectomy

**Table 3:** Test characteristics of FF-OCT on pancreatic tissue. True positives are FF-OCT images which were correctly identified as malignant. True negatives are FF-OCT images which were correctly identified as benign. False positives are FF-OCT images which were incorrectly identified as malignant. False negatives are FF-OCT images which were incorrectly identified as benign. Pathologist 1 is the experienced pathologist, pathologist 2 is the pathologist in training.

Pathologist	True positive	True negative	False positive	False negative	Not inter-pretable	Sensitivity	Specificity	Accuracy
1	28	47	8	11	6	72%	85%	80%
2	30	37	21	12	0	71%	64%	67%
Overall (mean)	29	42	15	12	3	72%	74%	73%

**Fig 3:**
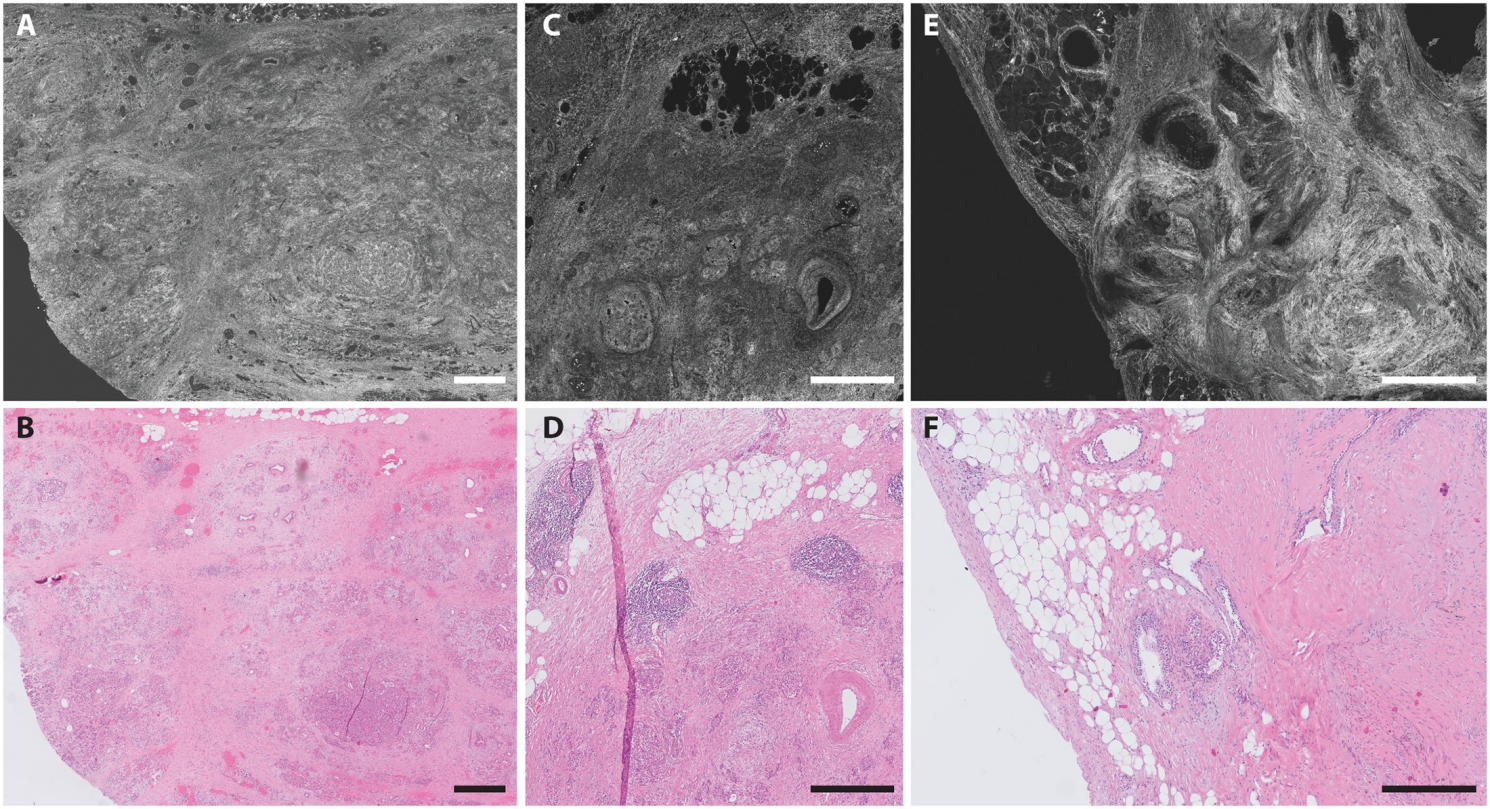
Example of benign pancreatic tissue FF-OCT images and corresponding histology. Fibrotic pancreatic tissue after neoadjuvant therapy (A-B), pancreatitis (C-D), and a serous cystadenoma (E-F). Scale bars all 500 μm.

**Fig 4:**
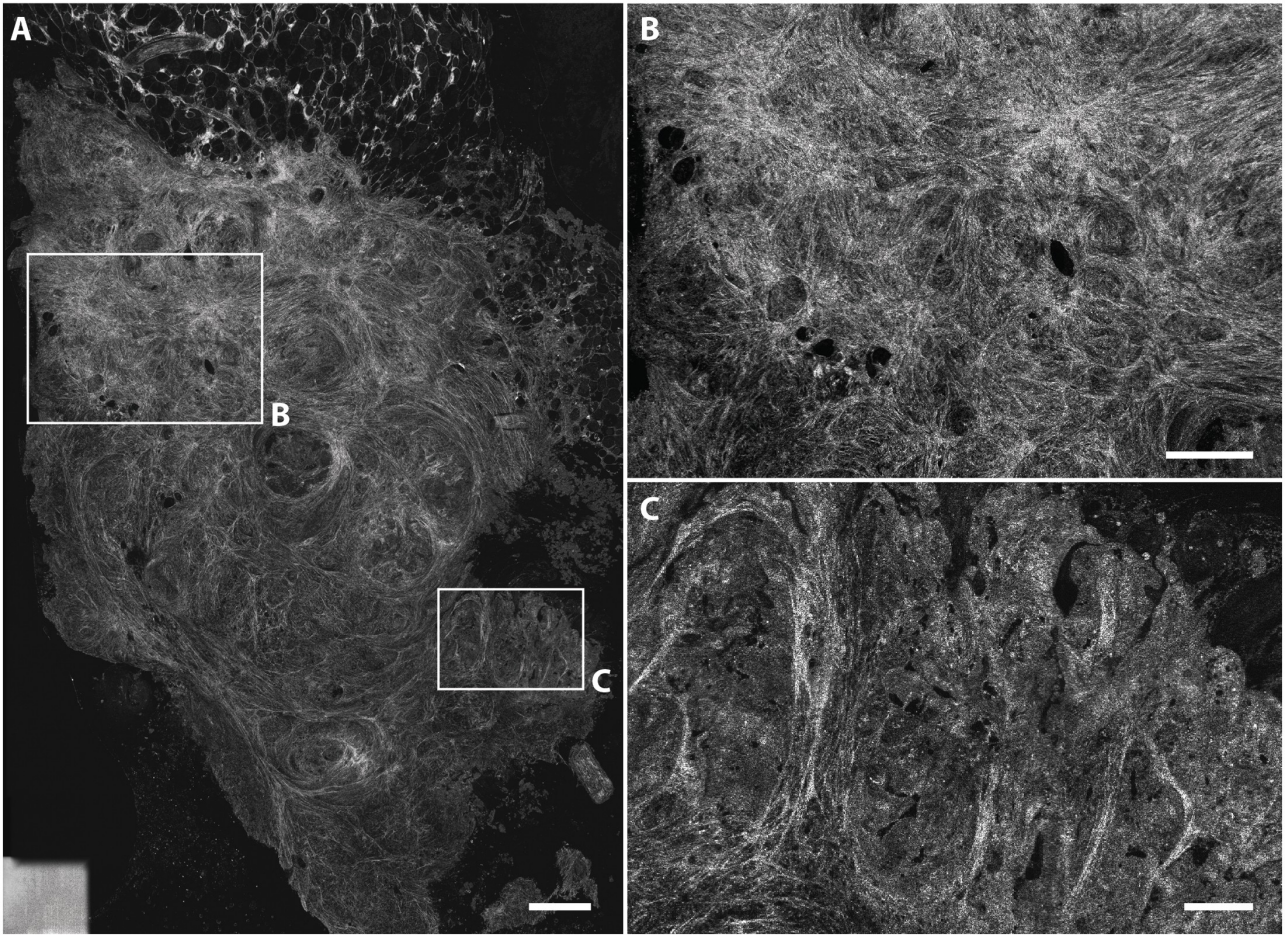
Example FF-OCT image of a well differentiated pancreatic ductal adenocarcinoma. A shows a n overview. B shows a magnified view of stromal disorganization, and C shows nests of tumor cells. Scale bars, 2 mm (A), 1 mm (B), and 500 μm (C).

A more detailed overview of the scores per histologic type is provided in Table 4. Normal pancreatic parenchyma was correctly recognised by both pathologists in 32 of the 40 cases. Atrophy and fibrosis (Fig 3) were wrongly scored by both pathologists in 63%, serous cystadenoma in 100% and neuroendocrine tumors in 25% of the cases. Pathologists evaluated 35 FF-OCT images of a PDAC (Fig 4), of which 34 were correctly scored as malignant by at least one pathologist. Well (grade 1) and moderately (grade 2) differentiated PDAC were scored as malignant by both pathologists in 67% and 57% of cases, respectively (Table 4). Whereas, poorly differentiated (grade 3) PDAC was scored as malignant by both pathologist in 1 out of 8 cases (13%).

**Table 4:** Accuracy per histological diagnosis.

	Correctly identified by both pathologists	Correctly identified by one pathologist	None correctly identified
Benign			
Normal pancreatic parenchyma	32	8	0
Pancreatitis	1	2	0
Atrophy or fibrosis	1	2	5
Serous cystadenoma	0	0	2
Mucinous cystic neoplasm	1	0	0
Malignant			
Grade 1 NET	0	1	1
Grade 2 NET	1	1	0
Grade 1 PDAC	4	2	0
Grade 2 PDAC	12	8	1
Grade 3 PDAC	1	7	0
Malignant IPMN	1		
Total^a^	**54**	**31**	**9**

^a^ 6 images were scored as not interpretable by one of the pathologists, these are excluded from this analysis.

The first 50 FF-OCT images given to the pathologists were taken from deparaffinised FFPE pancreatic tissue specimens, whereas the others were taken from fresh specimens. Univariate analysis showed no significant difference (P = 0.24) in evaluating fresh or deparaffinised FFPE FF-OCT images, however, fresh FF-OCT images appear to provide a more detailed view and improved visualization of tumor stroma.

### Interobserver variability

**Fig 5:**
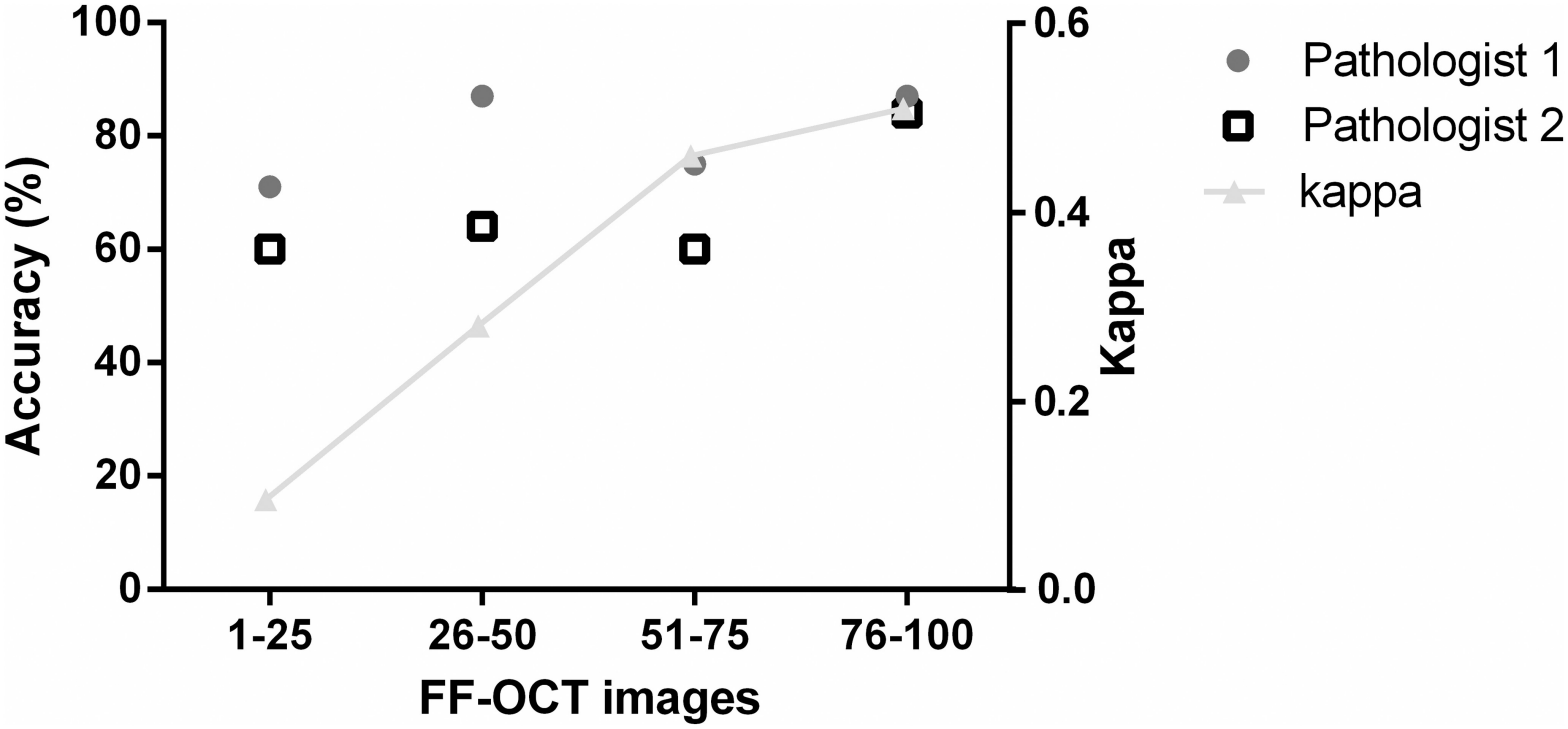
Accuracy and interobserver variability. Pathologist 1 is the experienced pathologist, pathologist 2 is the pathologist in training.

The overall interobserver variability (Kappa) was calculated and there was a fair agreement between the pathologists (Kappa: 0,33). After presentation of each set of 25 consecutive FF-OCT images the interobserver variability was measured and an increase of 0.1 to 0.5 was observed, as shown in Fig 5.

## Discussion

In this study we investigated whether pathologists were able to distinguish malignant from benign pancreatic tissue based on FF-OCT images. We found that normal, healthy pancreatic tissue could be distinguished from malignant tissue very well due to the characteristic architecture of normal pancreatic parenchyma. Distinguishing other benign tissue types like chronic pancreatitis was harder. We also found that the experienced pathologist was better in assessing the FF-OCT images than the pathologist in training. To our knowledge this is the first study to evaluate the correlation between H&E slides and FF-OCT in detecting malignancies in fixated and fresh pancreatic resection specimens. The diagnostic accuracy of 73% obtained in this study is not yet adequate, but we believe this can be improved upon by taking several measures: using other contrast mechanisms, improving knowledge on collagen topology, and more extensive training.

Pancreatic pathology and in particular morphological differentiation between pancreatic ductal adenocarcinoma and pancreatitis is challenging even on H&E. On FF-OCT this differentiation is difficult as well; although FF-OCT provides high resolution, the endogenous contrast is not proficient to see cell nuclei. Techniques to reveal subcellular metabolic contrast are being developed, showing encouraging results [30]. Other contrast mechanisms can provide additional information and thereby increase diagnostic accuracy.

Secondly, improved knowledge on the collagen topology could increase the diagnostic accuracy. As collagen reflects light very well, tumor stroma is evidently visible on FF-OCT images. The pathologists mostly evaluated the FF-OCT images based on these architectural features. However in this study, both pathologists often misdiagnosed pancreatic atrophy and fibrosis as malignant. In these cases the presence of collagen fibers dominating the image could be mistaken for tumor stroma as PDAC is characterized by extensive and disorganized desmoplastic stroma [31]. Recently it was shown that its collagen topology differs significantly from that of pancreatitis [32]. Improved knowledge on the collagen topology of these benign conditions, and possibilities to detect these differences and correlation between stromal alignment and organization, and diagnosis, could increase the diagnostic accuracy. This feature could also be exploited in the future by automated detection of collagen characteristics, as was previously done for second harmonic generation images of breast cancer [33].

Our study consisted of two parts: 50 image regions of deparaffinised tissue and 50 image regions of fresh tissue shown to the pathologists in that order. In tissue imaged both fresh and deparaffinised we see that the intensity of collagen is higher in fresh tissue (S1 Fig). We did not find significant differences in the assessment between these parts. However, an increase in accuracy was shown for both pathologists in the second half (images 26–50 and 76–100) of each part, suggesting a learning curve. Another study on FF-OCT where they looked at prostate biopsies showed an overall accuracy of 70%, with a learning curve from 60% to 80% after evaluation of 119 images; this is concordant with our data [34]. A later study on FF-OCT images of prostate core biopsies showed, after extensive training an overall accuracy of 93%. This implies that for pancreatic tissues further improvement is possible [35], therefore we believe that more training could further improve the diagnostic accuracy. We made a flowchart for assessment of pancreatic FF-OCT images which could support pathologists, although it should be validated in future studies (Fig 6).

**Fig 6:**
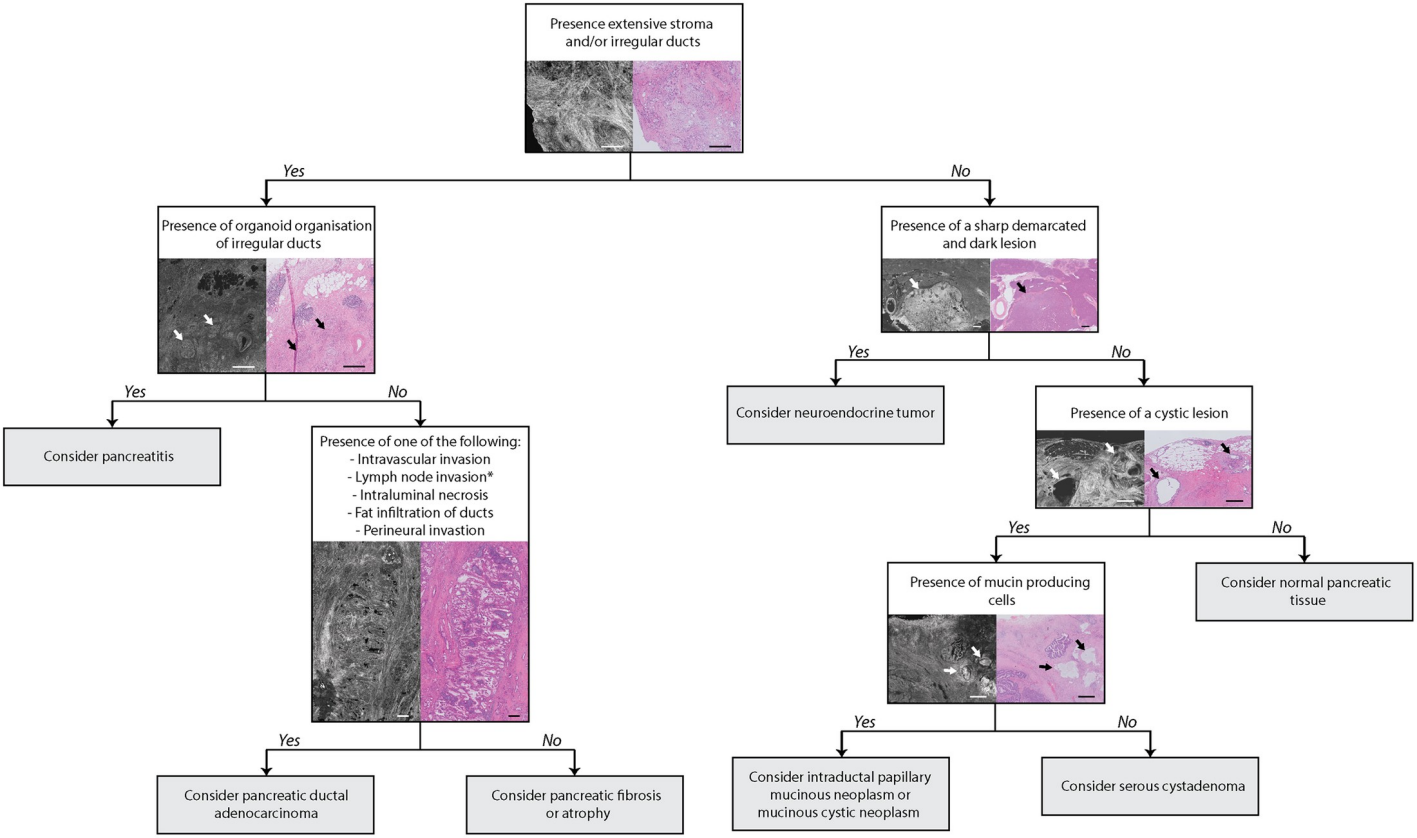
Proposed decision tree to evaluate pancreatic FF-OCT images. Scalebars are all 500 μm. *The FF-OCT image and corresponding histology image only depict lymph node invasion.

Finally, we did not provide any clinical data to the pathologists. This lack of clinical information made interpretation even more difficult as compared to daily practice when such information is available and taken into account in the interpretation of the histomorphology.

We have not imaged resection margins of the surgical specimens as that would have affected the clinical workflow too much, however, we included fresh pancreatic tissue for imaging to mimic the future application of intraoperative use. Moreover, we selected interesting and clinically relevant locations on the H&E and the corresponding FF-OCT images; for example, transition zones of infiltrating tumor glands and surrounding tissue (S2 Fig). Furthermore, to achieve an R0 resection margin in Europe, imaging up to 1 mm is necessary. In this study, we imaged at a depth of 20 μm to ensure good correspondence with reference H&E. As resolution decreases with increasing imaging depth, future studies should analyze the influence on accuracy with increased imaging depth.

The more experienced pathologist did not find the imaging quality good enough to give a diagnosis in six cases that were excluded from further analysis. These images were equally distributed between the different groups (malignant, benign, fresh, deparaffinised) we believe that this exclusion does not represent a specific bias.

Several other groups tried to identify pancreatic tumor tissue in order to reduce the amount of positive margins during surgery using various techniques. Hu et al. [36] used nonlinear optical microscopy to image pancreatic tumor xenografts harvested at different stages. They also saw an increased density of the collagen fibers in tumor compared with normal tissue. However, they did not do a blind reading of the images. Eberlin et al. [37], used mass spectrometry imaging and an automatic classifier to distinguish normal from cancer human pancreas tissue. They obtained a high agreement with pathology, but excluded cellular compositions that were not accounted for in their classifier, such as inflammation and necrosis, which is in contrast with our study, which included a broad spectrum of benign areas of disease to mimic clinical scenarios. Also our group studied the use of fluorescence-guided surgery during pancreatic resections. However, in human studies the results have been disappointing so far, as no useful tumor demarcation could be visualized with non-specific contrast agents [38, 39]. Currently, studies using tumor-specific fluorescent contrast agents are ongoing (Trial ID:NTR5673) [40].

Resection margins can either be examined *in vivo* before and during resection and after resection in the resection bed, or *ex vivo* on the resected specimen. Erickson-Bhatt et al. [41], used a portable OCT system to image the resection bed after a wide local excision of breast cancer. Tao *et al*. [42], also looked at resection margins in breast cancer, but examined *ex vivo* frozen sections using nonlinear microscopy, reaching an accuracy of 94.1%. We envision that FF-OCT could eventually be used during surgery at clinically suspect resection margins to further improve radical resection rates by extending the resection margin to facilitate *en bloc* tumor removal or by resecting additional tissue after suggestion of residual disease.

The FF-OCT device used in this study is not yet applicable in surgery. For translation into the operating room for *in vivo* imaging a handheld and faster device is necessary; for *ex vivo* assessment of resected specimens a bench top system is sufficient. Progression is made on both accounts. The first handheld endomicroscope based on FF-OCT was recently described by Benoit a la Guillaume et al. [43], which opens new perspectives for *in vivo* imaging. Furthermore, a 7 times faster and 3 times more sensitive camera was introduced in the device, also bringing it closer to clinical implementation [44].

In conclusion, FF-OCT could distinguish normal pancreatic tissue from pathologic pancreatic tissue, however, further development of the FF-OCT device and more experience in evaluating FF-OCT images of the pancreas is necessary before introduction in the clinical practice of pancreatic surgery.

## Supporting information

S1 Fig Influence of tissue processing on FF-OCT image quality. A pancreatic adenocarcinoma scanned freshly (A), after formalin fixation (B), and after deparaffinisation (C), with the corresponding H&E image (D). Scale bars all 500 μm.
(TIF)

S2 Fig An example of a transition zone of a well-differentiated pancreatic ductal adenocarcinoma and surrounding normal tissue. The FF-OCT image (A), inversed FF-OCT image (B), and the corresponding H&E image (C) are shown. The arrow shows normal pancreatic tissue and the arrowhead marks malignant glands. Scale bars all 500 μm.
(TIF)

S1 File Supporting data. (ZIP)
