# Supplementary figures and images for: Validation of full-field optical coherence tomography in distinguishing malignant and benign tissue in resected pancreatic cancer specimens
Source: PLoS One. 2017 Apr 17;12(4):e0175862. doi: 10.1371/journal.pone.0175862 (PMC5393621; doi:10.1371/journal.pone.0175862)

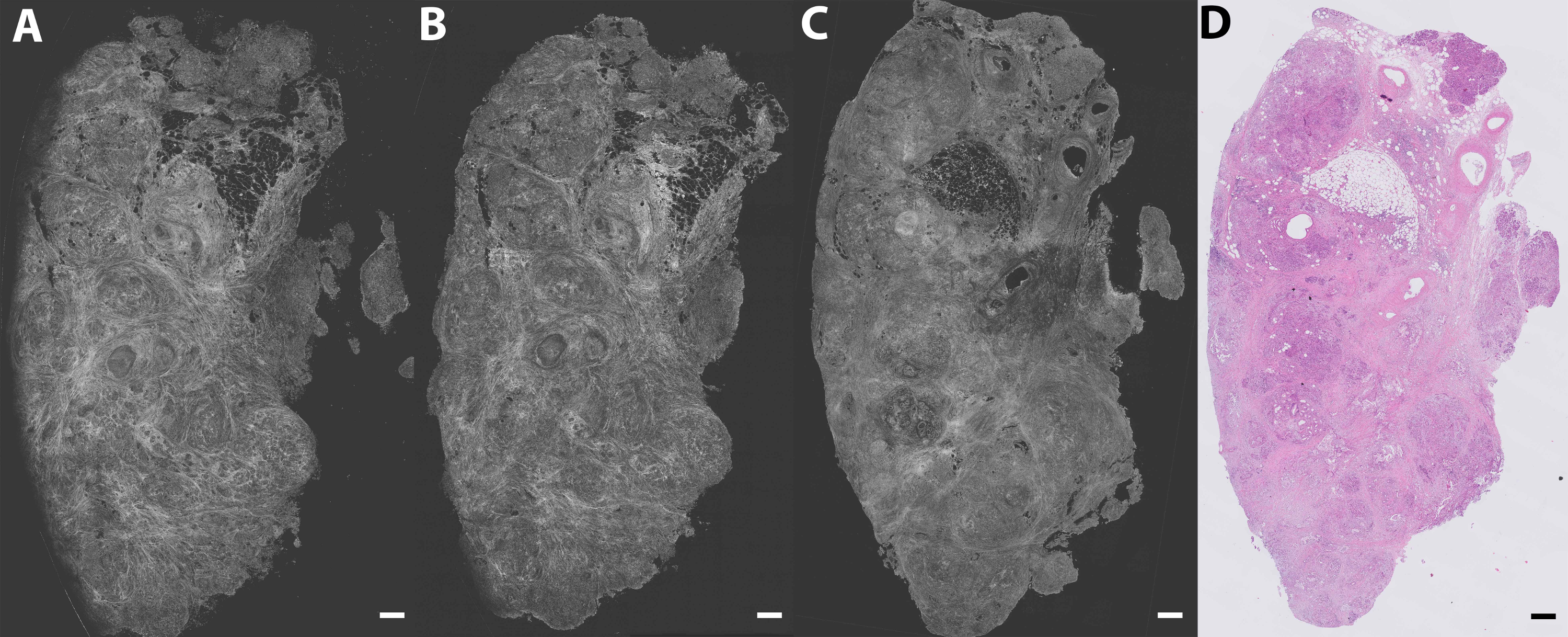

Supplement: S1 Fig — A pancreatic adenocarcinoma scanned freshly (A), after formalin fixation (B), and after deparaffinisation (C), with the corresponding H&E image (D). Scale bars all 500 μm. (TIF) [file pone.0175862.s001.tif]

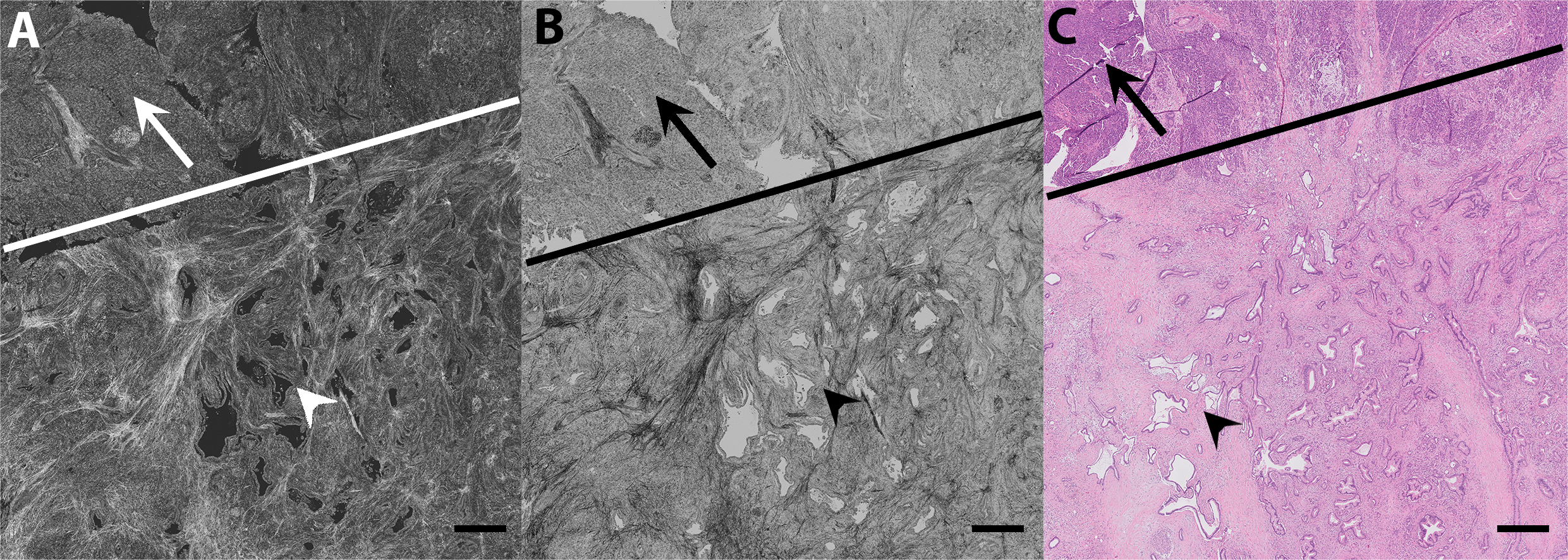

Supplement: S2 Fig — The FF-OCT image (A), inversed FF-OCT image (B), and the corresponding H&E image (C) are shown. The arrow shows normal pancreatic tissue and the arrowhead marks malignant glands. Scale bars all 500 μm. (TIF) [file pone.0175862.s002.tif]
